# Functional Outcomes After Discectomy for Recurrent Lumbar Herniation Using the Destandau Endospine System: A Retrospective Study of 44 Patients

**DOI:** 10.7759/cureus.49753

**Published:** 2023-11-30

**Authors:** Paresh C Dey, Saurav N Nanda, Saswat Samant, Ashok Gachhayat

**Affiliations:** 1 Orthopaedics, Kalinga Institute of Medical Sciences, Bhubaneswar, IND

**Keywords:** revision surgery, destandau endospine system, recurrent disc herniation, interlaminar discectomy, endoscopic discectomy

## Abstract

Background

Recurrent disc herniation is a major cause of morbidity and surgical failure after disc surgery. This study aimed to investigate the feasibility, safety, and effectiveness of the Destandau endospine system (DES) for treating recurrent lumbar disc herniation (LDH).

Methodology

A total of 44 patients who underwent minimally invasive Destandau endoscopic lumbar discectomy (DELD) for recurrent LDH were included in this study. All data were collected retrospectively. The preoperative and postoperative visual analog scale (VAS) score was used for the evaluation and gradation of pain. The clinical outcome was analyzed according to modified MacNab criteria. The minimum follow-up was two years. Preoperative and postoperative VAS scores were compared using the paired Student’s t-test. Statistical significance was set at a p-value <0.05.

Results

The mean surgical time was 30 ± 20 minutes. The VAS score for leg pain was improved in all cases from 5.9 ± 2.1 to 1.7 ± 1.3 (p< 0.001). In 98% of cases, a successful outcome was noted (excellent or good outcome according to MacNab criteria). In three (7%) patients, incidental durotomy occurred, but there was no neurological worsening, cerebrospinal fluid fistula, or negative influence on the clinical outcome. No recurrence or instability occurred in our series.

Conclusions

The clinical outcomes of minimally invasive DES for LDH were found to be comparable with the reported success rates of other minimally invasive techniques reported in the existing literature. The dural tear rate was independent of postoperative morbidity and functional outcome. The technique is a safe and effective treatment option for recurrent LDH.

## Introduction

Recurrent disc herniation is noticed in 5-11% of cases following disc excision and represents a major cause of morbidity and surgical failure [1]. One meta-analysis estimated that the prevalence of recurrent disc herniation after percutaneous endoscopic interlaminar discectomy (PEID) and percutaneous endoscopic transforaminal discectomy (PETD) is 4.2% and 3.4%, respectively [2]. The recurrent disc herniation rate by Destandau endoscopic lumbar discectomy (DELD) in our previous study was 0.3%, and the overall reoperation rate in the Kaushal and Sen study was 5% [3,4].

Recent literature suggests a high success rate of revision surgery for the management of recurrent disc herniation [5,6]. However, there is a lack of consensus regarding the ideal surgical procedure for recurrent lumbar disc herniation (LDH). There is controversy regarding the advantages of adding fusion along with discectomy during the revision surgery [7]. The conventional open technique for discectomy for recurrent disc herniation requires wide exposure and may need an additional procedure such as lumbar interbody fusion [8,9]. Patients undergoing interbody fusion surgery are more prone to developing adjacent segment disease [10].

Although the literature supports repeated discectomy for recurrent disc prolapse, it is associated with complications and poor results. Repeated discectomy becomes more difficult because of scar tissue. There is an increased chance of bleeding, dural tear, and nerve injury. It may also increase the risk of segmental instability due to further removal of posterior structures, including facet joints [1]. Recently, many percutaneous endoscopic procedures have been developed to perform minimally invasive surgery to treat LDH with results comparable to the conventional open technique [2-4].

Even in recurrent disc herniation, the newer endoscopic technique is equally effective in comparison with open and microsurgery without compromising bony stability and can avoid the adjacent level pathology by avoiding the fixation [1,7-9,11]. Ruetten et al. mentioned that the revision surgery can be conducted after percutaneous endoscopic discectomy as the index operation using the full endoscopic transforaminal and interlaminar discectomy [10,12-14]. However, there is a paucity of literature on the outcomes of endoscopic discectomy following open discectomy as the index operation for recurrent LDH [15,16].

Over the last decade, microendoscopic discectomy (MED), PETD, and PEID have been applied to treat recurrent LDH [17-22]. The endoscopic lumbar discectomy (DELD) procedure using the Destandau endospine system (DES) for recurrent disc herniation has not been reported in the literature to date. We designed this study to investigate the feasibility, safety, and effectiveness of DES for the surgical management of recurrent LDH.

## Materials and methods

**Figure 1:**
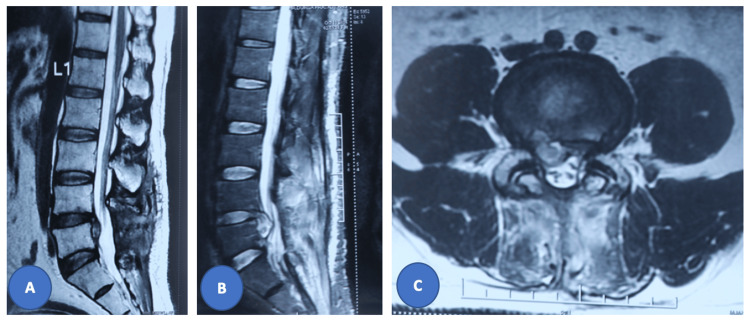
Preoperative MRI images of the index surgery showing right paracentral disc extrusion and caudal extension at the L4-5 disc causing severe canal stenosis. (A) T1 images of the sagittal section; (B) T2 images of the sagittal section; (C) axial images at the L4-5 level.

**Figure 2:**
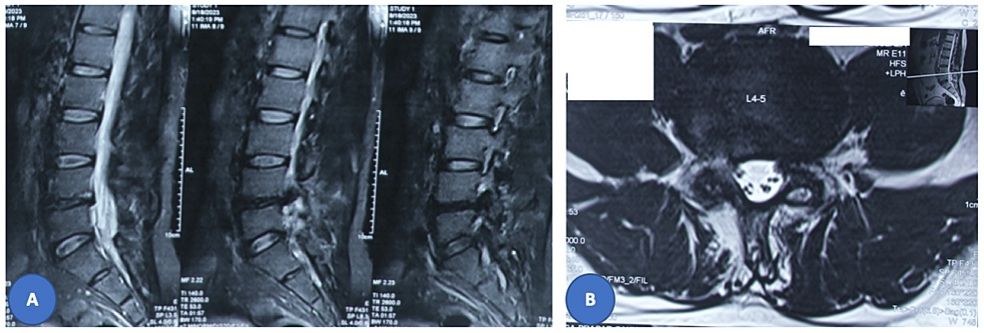
Preoperative MRI images of revision surgery showing the L4-5 level laminectomy defect and left paracentral disc extrusion contiguous with the posterior disc margin suggestive of recurrent herniated disc. (A) sequential T2 images of the sagittal section; (B) axial images at the L4-5 level.

Approval was obtained from the Ethics Committee of Kalinga Institute of Medical Sciences, Bhubaneswar, India (approval number: 121/ORTHO/2021). The institutional review board approved the waiver of consent for this study. We included 44 patients (26 male and 18 female), with ages ranging from 25 to 62 years (mean 38 years), with recurrent disc herniation who underwent minimally invasive DELD (Figures 1, 2).

We performed the endoscopic discectomy by DES using the paramedian interlaminar approach under general anesthesia by a single surgeon (PC) between August 2010 and August 2021 at a tertiary healthcare institute. The duration of the symptom-free interval after a previous surgery was eight months to 11 years. There were 24 (55%) patients with pathology at L4-5, 12 (27%) patients with pathology at L3-4, four (9%) patients with pathology at L5-S1, three (7%) patients with pathology at L2-3, and one (2%) patients with pathology at L1-2 level. Radicular pain was greater on the right side in 17 (39%) patients and greater on the left side in 27 (61%) patients. We noted motor weakness in six (14%) patients, sensory deficit in 11 (25%) patients, and loss of reflexes in 22 (50%) patients. The time period from the onset of symptoms to surgery varied from six weeks to eight months.

The inclusion criteria included patients with recurrent disc herniation with symptoms of pain, increased intensity of pain, radiculopathy, and loss of sensation and/or paresthesia along the dermatome with loss of motor strength and/or reflex changes in the correspond­ing muscle. The recurrence of disc herniation with compression of the nerve was confirmed with contrast MRI. Recurrent disc herniation after open lumbar discectomy, microdiscectomy, and discectomy by DES was included in our study. Only patients who failed a trial of conservative treatments were recruited. Patients with recurrent prolapsed intervertebral disc with cauda equina syndrome, associated with canal stenosis, isolated back pain, bilateral leg pain, and documented instability of the spine having more than grade 1 listhesis (Meyerding grading) were excluded. We also omitted the first two recurrent disc herniations operated by DES in view of the learning curve of the procedure.

All surgeries were performed using DES (Karl Storz, Tuttlingen, Germany). Details of this system, the position of the patient, localization of the surgical field, and incision were mentioned in our previous article [3]. The surgical technique for recurrent disc surgery is tricky due to the alternation of anatomical structures. The foremost thing to identify is the remaining bony structure. Soft tissue was dissected from the remaining bony structure. A curette was helpful in dissecting soft tissue and identifying the bone and fibrous tissue junction. Gradually and cautiously, the cleavage was made between bone and fibrous tissue. If laminae were present, they were removed to expose the virgin dural sac. However, most of the time, in open discectomy lamina was absent. Hence, mostly, the target was facet and undercut the medial part of the available facet till the lateral edge of the dura and traversing nerve root.

The scar tissue over the dura was left untouched to ensure strong adhesion. The nerve root was identified and decompressed until the neural foramen. That was the lateral recess adequate decompression, and this is an advantage of this system. The coexisting lateral recess stenosis decompressed easily without losing segmental stability and at the same time was getting good space laterally for disc identification. Recurring fragments were covered with strong fibrous tissue which made it difficult to identify the disc space. In a few cases, the c arm was used to identify the disc space. A cottonoid was passed at the lateral edge of the dura at the shoulder of the traversing root cranially to retract them medially. This was a good technique to create a space laterally and was helpful for the identification and removal of the recurrent disc material.

Once the adequate discectomy was done and complete nerve root decompression was achieved, the surgical field was irrigated with a copious amount of saline, and the wound was closed by three or four subcuticular vicryl 3-0 sutures. In the end, a waterproof skin dressing was applied. Patients were allowed to be out of bed on the same day after recovery from anesthesia and were discharged on the first or second postoperative day as they became comfortable. They were gradually allowed to return to work by two to three weeks but were told to refrain from heavy manual labor for at least four to six weeks postoperatively.

Data were entered in Microsoft Excel (Microsoft Corp., Redmond, WA, USA), and analysis was done using Stata 12 (StataCorp., College Station, TX, USA). Prevalence was reported as a proportion with a 95% confidence interval. Differences among proportions were calculated using the chi-square test. Scores were represented as median and interquartile range (IQR). Differences in means were calculated by the t-test. P-values less than 0.05 were considered significant.

## Results

In our study, we included 44 patients (26 male and 18 female), with ages ranging between 25 and 62 years old (mean 38 years), with recurrent disc herniation who underwent minimally invasive DELD. Demographic details of the patients are presented in Table 1. The average surgical time was 30 ± 20 minutes. The average fluoroscope exposure for localization of level was four shoots (between two and seven shoots). The mean blood loss was 40 ± 15 mL as measured from the volume in the suction chamber.

**Table 1:** Demographic details of sample populations.

Variables	Parameters	Number of cases
Age group	25 years to 62 years	
Gender	Male	26 (59%)
	Female	18 (41%)
Site of pathology	L 1-2	1 (2%)
	L 2-3	3 (7%)
	L 3-4	12 (27%)
	L 4-5	24 (55%)
	L 5-S1	4 (9%)
Radicular pain	Right side > left side	17 (39%)
	Left side > right side	27 (61%)
Neurological deficit	Motor weakness	6 (14%)
	Sensory Loss	11 (25%)
	Loss of reflexes	22 (50%)
Time gap (from the onset of symptoms to intervention)	6 weeks to 8 months	

The patients were followed up for a minimum of 24 months. There was no neurological worsening. The visual analog scale (VAS) for the leg and back was reduced to 1.7 ± 1.3 from 5.9 ± 2.1 (p < 0.001).

In the two-year follow-up, 44 (100%) patients were relieved of radiculopathy (leg discomfort), and 36 (82%) patients were relieved of back pain and were satisfied with the procedure. According to modified MacNab criteria, the results were excellent in 39 (89%) patients, good in four (9%) patients, fair in one (2%) patient, and poor in none of the patients (0%). Therefore, 98% showed excellent to good outcome.

**Figure 3:**
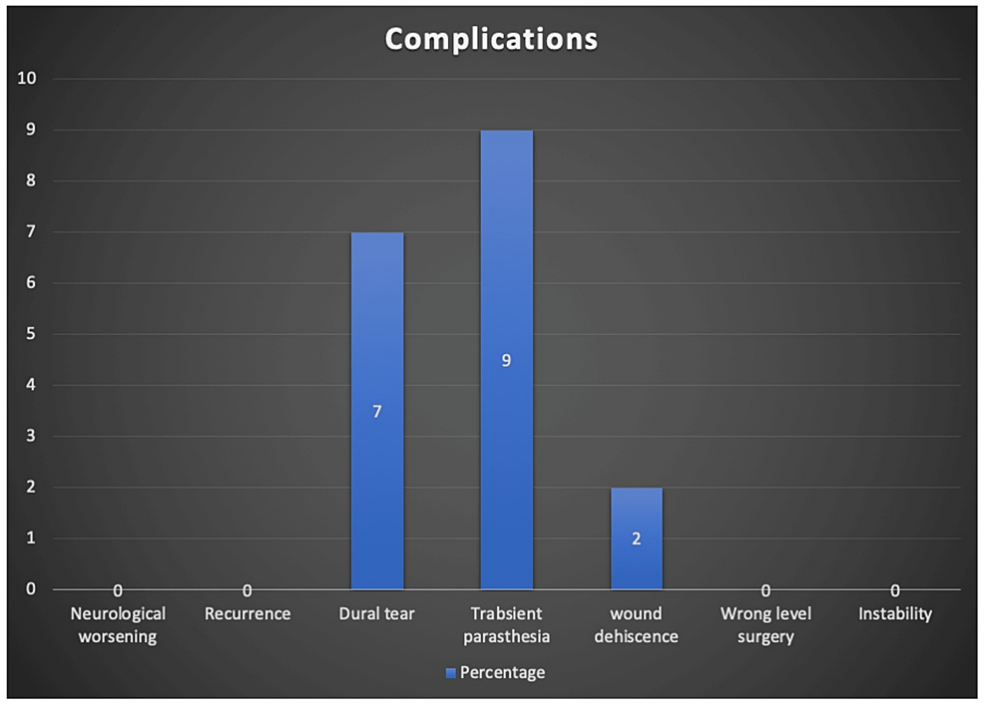
Complications.

There were no serious complications such as neurological deficit or cauda equina syndrome, and the overall complication rate was 0%, as depicted in Figure 3. No recurrence has been noted yet. Three (07%) patients had a dural tear, managed with a muscle patch and surgicel. All these dural tear cases were monitored in the hospital for four days, and all healed without further complications. Four (9%) patients complained of paresthesia, which subsided gradually with oral medication in six weeks. Mild muscle weakness (4/5 MRC grading) of extensor hallucis longus was noted in four (9%) patients, and their strength recovered within six weeks. Wound dehiscence was found in one (2%) patient. She was obese, and resuturing was done. The wound healed uneventfully in two weeks. There was no wrong-level surgery in our series. None of the patients developed disc space degeneration or instability (Figure 3).

## Discussion

DES is a mobile operating tube that can be easily wended into all nooks and corners according to the needs of the surgeon. Unlike other tubular systems, it does not require any fixation of the device at a particular position or further readjustment, making it user-friendly and saving surgical time. In our series, due to the above-mentioned factors, the average surgical time was 30 ± 20 minutes, which is substantially less than other series using open, microscopic, MED, PETD, and PEID [1,17,20]. The overall success rate in our DES series was 98% and is comparable to the reported success rate of open, microscopic MED, PETD, and PEID [1,4,17,20].

Revision spinal surgery is more challenging than primary surgery. There is always a high risk of dural tear and nerve injury in revision surgery due to the presence of scar tissue [1,20]. Ebeling et al. mentioned a complication rate of 13% in their study after repeated discectomy by open surgery, with dural tears or infections being the most common problems [23,24]. In the endoscopic interlaminar (PEID) series [1], fewer than 10% of patients had complications such as dural tears and postoperative dysesthesia. There were no neurological complications in our series.

Open surgery involves an extended dissection with muscle splitting, laminectomy, and facetectomy. This may lead to segmental instability and the persistence of postoperative low back pain [3,10,22]. In PELD, either by transformational or interlaminar, the resection of posterior stabilizing structures such as the lamina, facet, ligament, and muscle is minimal. Hence, the rate of complications such as instability is low [1,25]. In our series, postoperative instability was not detected.

As reported by Ruetten et al., the resection of intradiscal materials is inadequate in the full endoscopic interlaminar approach, as the level of interlaminar window and intervertebral space are divergent, which resulted in recurrent disc herniation in 6% of their cases [13,14]. This problem was overcome by subsequent PELD by widening the narrow interlaminar window using a specifically designed micro-osteotome for complete resection of intradiscal materials [1].

In our study, we found that DES could be utilized comfortably for recurrent disc herniation. Ruetten et al. and Kyung et al. mentioned that a full endoscopic operation achieves similar functional outcomes to those of microscopic open discectomy [1,10-14]. In our study, there was no significant complication or operation-related neurological deterioration. The minimally invasive DES is another surgical technique that can be useful in the treatment of recurrent disc herniation after microdiscectomy, DES, or an open primary discectomy.

In our series, similar to other endoscopic techniques, there are advantages over conventional surgery, such as improved intraoperative visualization of the surgical field and illumination, reduced surgical dissection, reduced blood loss, epidural edema (responsible for epidural scarring), less surgical time, and high patient satisfaction [1,12-14]. The limitations of this study are that the procedure has a steep learning curve and involves the use of specific instrumentation.

## Conclusions

Our study shows that an optimum neural decompression and discectomy is possible using DES for recurrent disc herniation after conventional open, micro, and DES discectomy. We conclude that DES approaches are possible and are effective and safe treatment options for recurrent LDH.
